# Ammonia Decomposition Catalyzed by Co Nanoparticles Encapsulated in Rare Earth Oxide

**DOI:** 10.1021/acs.jpclett.4c03309

**Published:** 2025-01-14

**Authors:** Hiroshi Mizoguchi, Shunqin Luo, Masato Sasase, Masaaki Kitano, Hideo Hosono

**Affiliations:** † Research Center for Materials Nanoarchitectonics (MANA), National Institute for Materials Science (NIMS), 1-1 Namiki, Tsukuba, Ibaraki 305-0044, Japan; ‡ MDX Research Center for Element Strategy, Institute of Science Tokyo, 4259 Nagatsuta, Midori-ku, Yokohama 226-8503, Japan

## Abstract

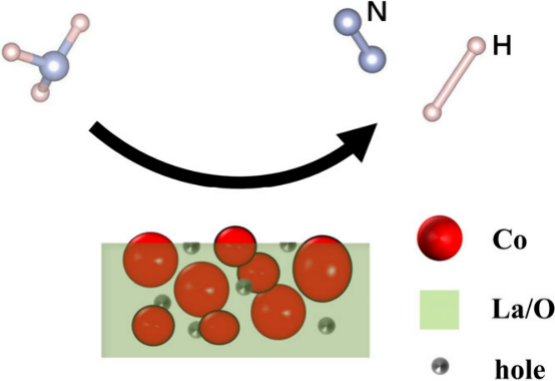

We fabricated Co-based catalysts by the low-temperature thermal decomposition of R–Co intermetallics (R = Y, La, or Ce) to reduce the temperature of ammonia cracking for hydrogen production. The catalysts synthesized are nanocomposites of Co/RO_*x*_ with a metal-rich composition. In the Co_13_/LaO_1.5_ catalyst derived from LaCo_13_, Co nanoparticles of 10–30 nm size are enclosed by the LaO_1.5_ matrix. The nanocomposite exhibited superior catalytic activity (91% at 500 °C), which was attributed to dual advantages; the low workfunction of the supporter, O-deficient LaO_1.5-x_ nanoparticles, promotes electron donation to the Co catalyst in the interface, which leads to enhanced N–H bond dissociation. Moreover, such a composite structure is effective in suppressing the grain growth of Co nanoparticles because the LaO_1.5_ layer works as a diffusion barrier against Co. The thermal decomposition of intermetallics is a new route for the facile synthesis of catalysts having an electronically active support.

For the realization of hydrogen economy, a technology for the transport of hydrogen should be established.^1a,1b^ As candidates for hydrogen carriers, compressed gaseous hydrogen in cylinders, liquid hydrogen, liquid ammonia (NH_3_), and organic hydrides have been investigated thus far. Among these, ammonia has the advantage of being easy to liquefy and transport in large volumes. However, the method of extracting hydrogen from ammonia at low temperatures has not yet been established.^2a−2c^ The cracking reaction of NH_3_ is endothermic with Δ*H*_298_ = 46.1 kJ/mol and is thermodynamically favorable at high temperatures and low pressures.^3^ However, at the equilibrium cracking fraction of 98% at 400 °C under 1 atm, the cracking reaction does not proceed without catalysts (see the equilibrium value in Figure 1b). A catalyst mainly works in two elementary reactions. One is the dissociation of N–H bonds in the adsorbed NH_3_ molecule. The other is the recombination of N adatoms generated on the surface of the catalyst to form N_2_. The activity of catalysts shows a volcano-type trend as a function of the metal (M)–N interaction in the periodic table with the apex of Ru. Although Ru exhibits excellent properties for this reaction,^4a,4b^ there are two drawbacks, namely, the high and unstable price of Ru and its low natural abundance, which make it difficult to meet market demands. Since the pressure of NH_3_ during NH_3_ decomposition is higher than that during NH_3_ synthesis, the peak for the volcano plot shifts toward the region of weak M–N interaction.^5^ Thus, Co and Ni, which are relatively inexpensive, are good candidates for Ru alternative catalysts. Although there are many reports regarding these 3d transition-metal catalysts,^2a,2b^ the process that works even at lower temperatures (below 300 °C) is essential for practical use. Since the support effect on NH_3_ decomposition catalysts is not well-understood as far as we know, we have explored the use of supports. Recently, we have reported that the Ni/CeO_2_ catalyst synthesized by the low-temperature thermal oxidation of the CeNi_5_ intermetallic (IM) shows high activity.^6^ Its unique microstructure, that is, the large Ni/CeO_*x*_ interface arising from the interlocking of Ni nanoparticles with the CeO_2_ framework, was attributed to its high catalytic activity. Here, we report the high catalytic activity of Co/LaO_1.5_ with the unique microstructure. The dispersed Co nanoparticles attached to the LaO_1.5_ grain boundary phase enhance the catalytic reaction. The grain boundary phase suppresses the grain growth of metallic Co particles at high temperatures.

**Figure 1:**
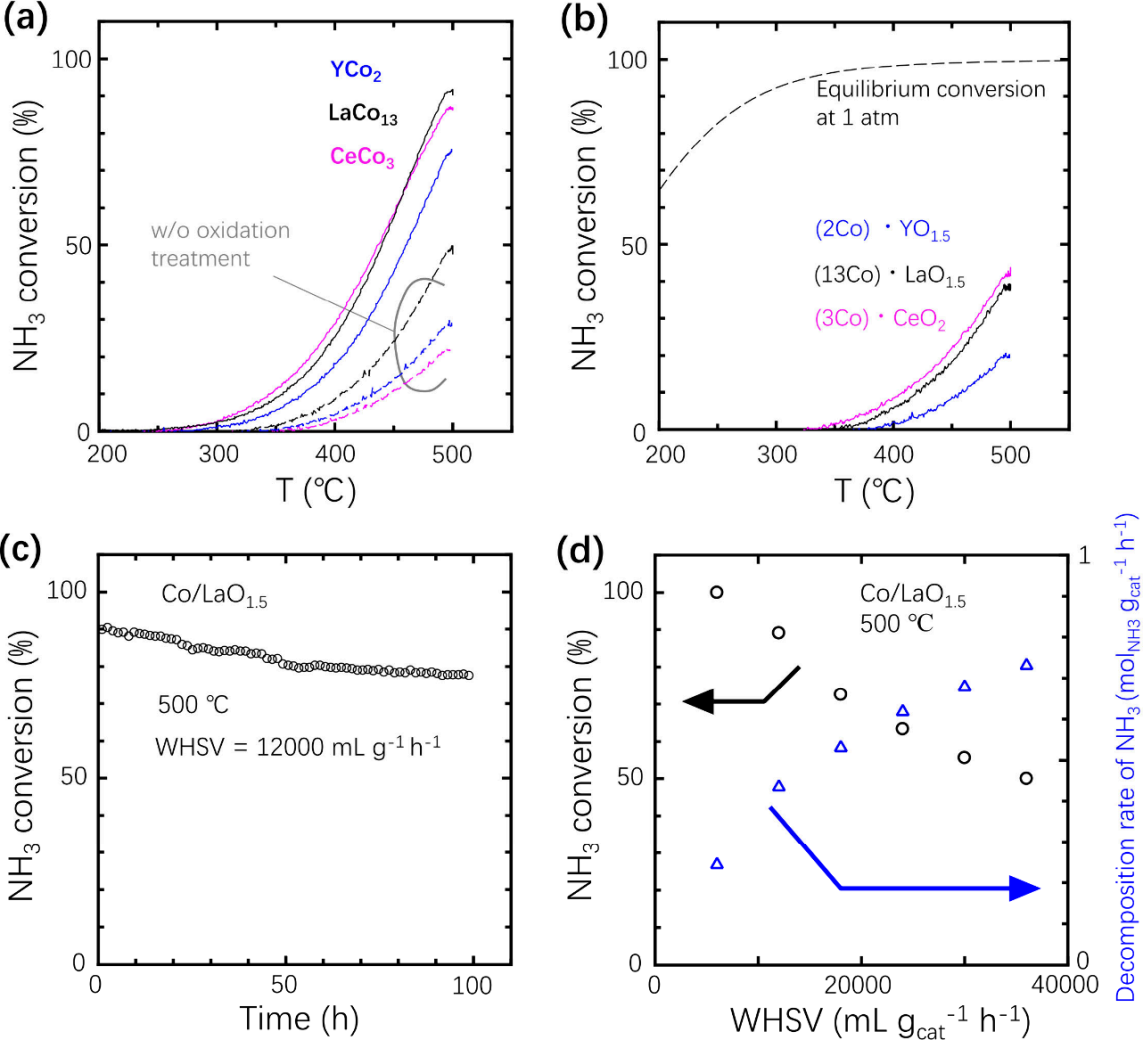
Temperature dependence of conversion in NH_3_ cracking over various Co-based catalysts at a weight hourly space velocity (WHSV) of 12000 mL_NH3_ g_cat_^–1^ h^–1^. Co-based IMs with (solid line) or without (dashed line) oxidation treatment. (b) Reference Co-based catalysts prepared by impregnation method (solid line) and calculated thermodynamically equivalent values (dotted line). (c) Time course of NH_3_ cracking over Co/LaO_1.5_ catalyst. (d) WHSV dependence of the conversion in NH_3_ cracking. As the WHSV increased, the activity decreased because of the shortening of the contact time between the catalyst and the NH_3_ molecule.

Many Co-based IMs decomposed in the catalytic activity test under NH_3_, resulting in the formation of Co particles that exhibit catalytic activity. The key to designing new catalysts is to induce Co nanoparticle formation via IM decomposition at the nanoscale level. Among the R–Co systems, we focused on IMs with R = Y, La, or Ce because of their electron donation power originating from the lower workfunction (WF) of R ions. There exist various IMs in an R–Co binary system. The pulverization of IMs is often possible because IMs commonly have mechanically brittle properties. This feature facilitates the compositional search for powder catalysts. As an example, the catalytic activity (conversion in NH_3_ cracking) for NH_3_ decomposition in the La–Co system is shown in Figure 2. The maximum catalytic activity was observed in La_7_Co_93_, that is, LaCo_13_. On the basis of the investigation of an R–Co binary system (R = Y or Ce), we also confirmed the high catalytic activities of CeCo_3_ and YCo_2_. Figure 1a shows the temperature dependence of the catalytic activity. The activity of LaCo_13_ or CeCo_3_ fabricated by the process shown in Scheme 1 became noticeable at ∼270 °C and reached 91 or 87% at 500 °C, respectively (solid lines in Figure 1a). Note that CeCo_3_ shows a catalytic activity of 30% at 400 °C. As described later, metallic Co particles are formed in these catalysts, which are responsible for the observed activity. Figure 1a also shows the data indicated by dashed lines for the catalysts without an oxidation treatment. The activities of these samples were increased by two or three times by oxidation treatment at 350 °C. Figure 1b also shows the activities of the Co/RO_*x*_ catalysts with the same cationic composition prepared by the impregnation method for comparison. Figure 1c shows the time course of the catalytic activity. The activity of LaCo_13_ at 500 °C gradually decreased to 78% in 100 h. Figure 1d also shows the gas flow rate dependence of the catalytic activity. Table 1 shows the apparent activation energies (*E*_a_ = 95–99 kJ/mol) of these catalysts, which were calculated from the Arrhenius plot. The observed *E*_a_ values suggest that these catalysts have the same rate-determining step. We compared the activities of these catalysts with those of Co-based catalysts reported thus far. Table S1 in Supporting Information summarizes representative reports on Co-based catalysts. We should compare these data carefully because the activity depends highly on the reaction temperature or weight hourly space velocity (WHSV). The obtained activities (25–30% at 400 °C) in Figure 1a are comparable to those of Co-based catalysts, which show the highest activities as previously reported (e.g., Co/Ba-promoted CeO_2_, Co/La-promoted MgO).^7^^8^^9^^10^

**Scheme 1:**
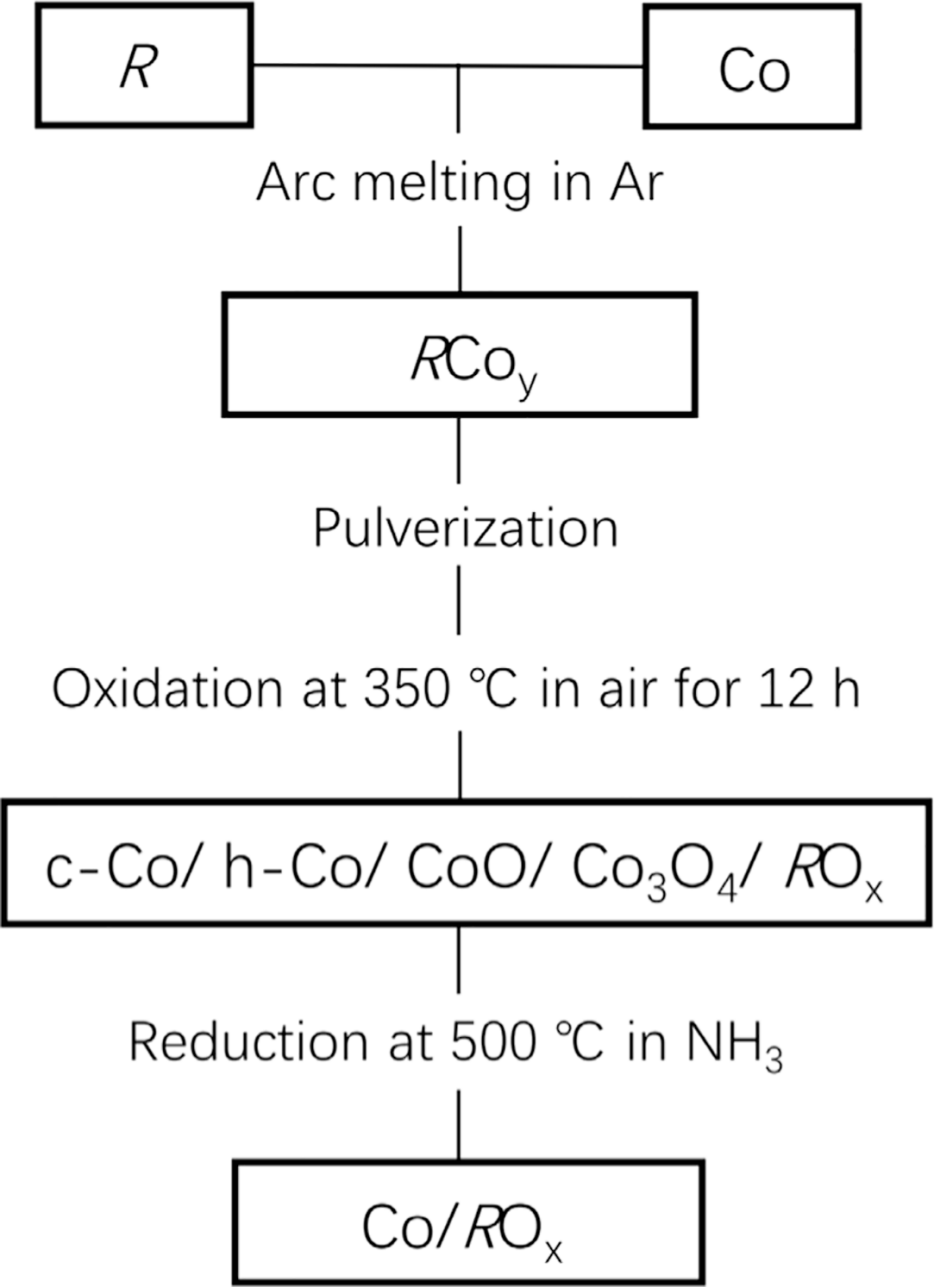
Flowchart for Preparing Co/RO_*x*_ Catalysts

**Figure 2:**
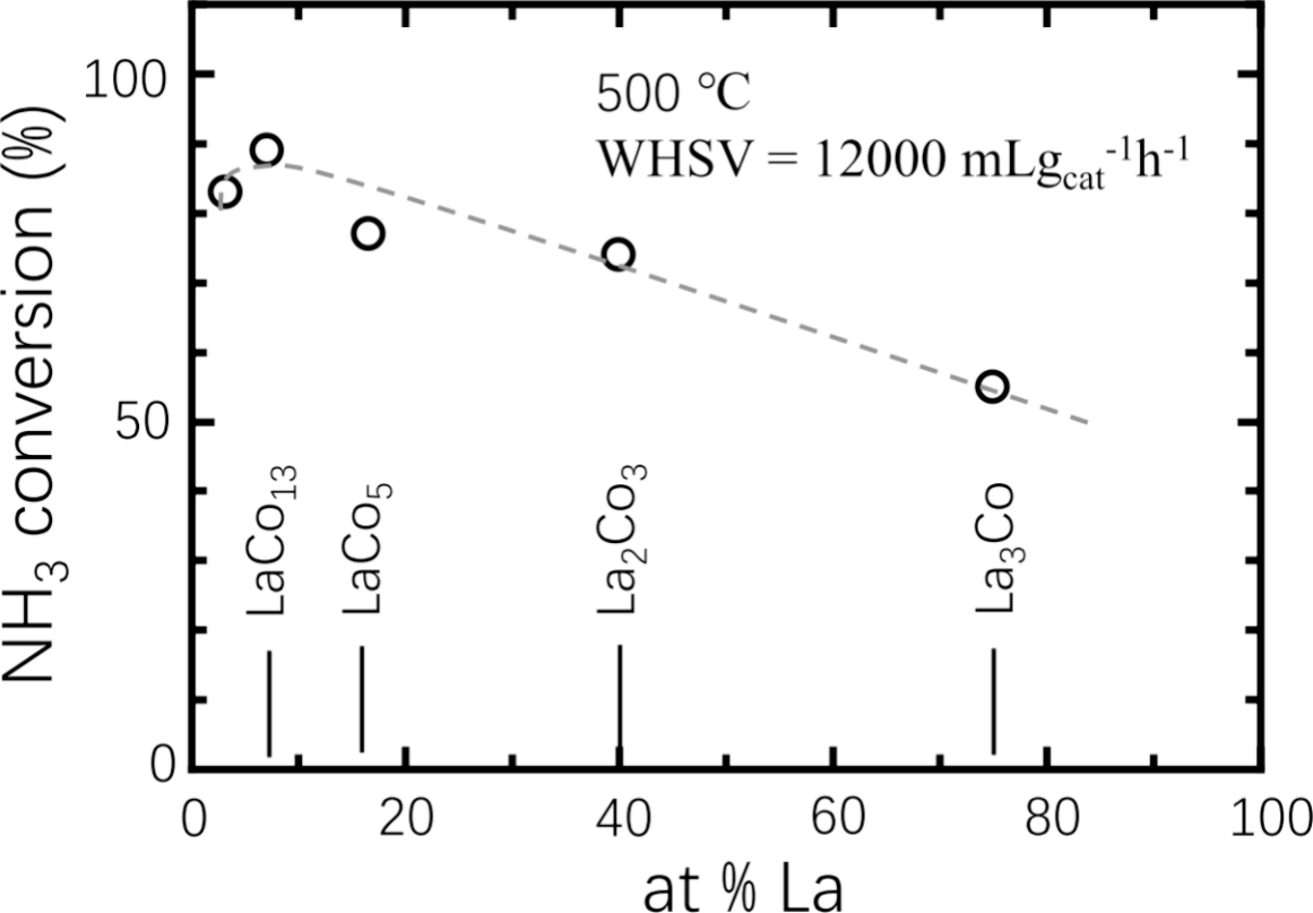
Chemical composition dependence in conversion of NH_3_ cracking for La–Co intermetallics.

**Table 1:** Properties of the Co-based catalysts after NH_3_ test

Catalyst	Conversion^a^.at 500 °C (%)	NH_3_ decomposition rate^a^ (mol_NH3_ g_cat_^–1^ h^–1^)	*E*_a_^b^ (kJ/mol)	Surface area (m^2^/g)	Co surface area (m^2^/g)	WF (eV)
Co/LaO_1.5_	90.5	0.43	99.2	4.8	0.52	4.7
Co/CeO_2_	86.7	0.42	94.8	15.2	1.68	5.2
Co/YO_1.5_	75.6	0.37	98.4	8.5	0.80	-
Co	-	-	-	-	-	5.3

a Reaction conditions: catalyst, 0.050 g; temperature, 500 °C; NH_3_ gas, 10 mL min^–1^, 12000 mL_NH3_ g_cat_^–1^ h^–1^; pressure, 0.1 MPa.

b Thermal activation energy (*E*_a_) was estimated in the temperature region of 310–370 °C.

The catalysts obtained were characterized to elucidate the origins of their activities. Figure 3a,b, respectively, shows the powder XRD patterns of LaCo_13_ and CeCo_3_ showing high activities. These IMs were decomposed into c-Co/h-Co/CoO/Co_3_O_4_/RO_*x*_ by oxidation treatment at 350 °C. After the NH_3_ test, Co oxides were converted into c-Co through reduction under NH_3_ and/or H_2_. Hereafter, LaCo_13_, CeCo_3_, and YCo_2_ synthesized by the procedure shown in Scheme 1 are indicated as Co/LaO_1.5_, Co/CeO_2_, and Co/YO_1.5_, respectively. The results indicate that the La–O affinity is stronger than the La–N or La–H affinity, as expected from the thermodynamic stability represented by the Ellingham diagram. We estimated the crystallite size (CS) of each phase appearing in these catalysts by a fundamental parameter method (Figure S1), and the estimated CSs are summarized in Table 2. Note that the sizes of RO_*x*_ phases were ∼10 nm, which were rather smaller than those of metallic Co particles, showing similarity with an inverse catalyst structure. SEM-EDX measurements indicated that the chemical composition of these catalysts agrees with that of the raw materials. Figure 4 shows the results of STEM observations of the Co/LaO_1.5_ catalyst. It also shows that the microstructure of the samples was uniform and that no large difference in microstructure was observed for different locations. The composition ratio of Co/La was determined as ∼13 by EDX, which was consistent with that of raw materials (LaCo_13_). Figure 4a,b and Figure 4c,d show the HAADF-STEM and EDX mapping images of the same region, respectively. These images indicate that the catalyst is a nanocomposite composed of metallic Co single crystals and a LaO_1.5_ matrix. Figure 4e shows the nanostructure observed by STEM. The Co nanoparticles of 10–30 nm size are enclosed by the LaO_1.5_ phase. The size of the Co particles was consistent with that estimated by Rietveld refinements. The HAADF-STEM image in Figure 4f shows the interface between a Co single crystal and LaO_1.5_. The LaO_1.5_ phase, whose lattice image is derived from the A-type lanthanide sesquioxide structure (trigonal, space group: *P*3̅*m*1), adheres closely to the Co single crystal. We see holes and hollows in the LaO_1.5_ phase, as shown in Figure 4a,b. We can also see the presence of the amorphous region of LaO_1.5_ in the interface. Such a nanostructure causes distortion and lattice defects in metallic Co, which is softer than LaO_1.5_. We also observed stacking faults at the center of the Co single crystal in the BF image, shown in Figure 4g. The theoretical ratio of lattice volumes V(26Co)/V(La_2_O_3_) = 77/23 was calculated from the unit cell volumes. The CS of LaO_1.5_ is inevitably small in the unique microstructure, and the CS value of ∼10 nm estimated from Rietveld refinements is consistent with the size (10–30 nm) of the Co particles and the theoretical ratio of lattice volumes. In the microstructure, separation of the matrix into the metallic Co and LaO_1.5_ phases occurs. Interdiffusion between these phases is restricted under low-oxygen-partial pressure atmosphere owing to the large difference in ionic size. That is why the LaO_1.5_ phase serves as a barrier for diffusion, preventing the grain growth of Co nanoparticles at high temperatures. We observed two polymorphisms of metallic Co. Phase transition occurs at ∼420 °C from the hcp-type crystal structure (low-temperature phase) to the fcc-type structure (cubic, high-temperature phase). The cubic phase is stabilized by surface energy, and nanoparticles tend to adopt the cubic polymorphism.^11^ As shown in Figure 3, the reduction of Co oxides under an NH_3_ flow leads to c-Co formation, indicating that the LaO_1.5_ phase contributes to the formation of the cubic phase by preventing the grain growth of Co particles. Thermal desorption spectroscopy (TDS) measurements provide H or N content information (Figure S2 in Supporting Information). Note that the obtained compositions of the Co/LaO_1.5_ and Co/CeO_2_ catalysts were Co_13_(LaO_1.5_)N_0.02_H_0.03_(H_2_O)_0.04_(CO_2_)_0.008_ and Co_3_(CeO_2_)N_0.16_H_0.02_(NH_3_)_0.02_(H_2_O)_0.05_(CO_2_)_0.05_, respectively. The behavior of N desorption from the Co/CeO_2_ catalyst was similar to that of Co_2_N.^12^Table 1 also summarizes BET surface areas and metal surface areas. Metal surface area of Co/LaO_1.5_ is ∼30% of that of Co/CeO_2_, in spite of the large Co content (Co/La = 13) in Co/LaO_1.5_. The XPS spectra of the Co/LaO_1.5_ and Co/CeO_2_ catalysts were collected in order to obtain information about the surface state. Figure S3a shows the La 3d spectrum of Co/LaO_1.5_. The peak at 834.9 eV was attributed to La 3d_5/2_ in La^3+^ state in oxide.^13^ We can see a weak shoulder at 832.5 eV, indicating reduced La ion (La^(3-x)+^). From the peak area, the ratio La^(3-x)+^/La^3+^ was calculated to be 0.32. In the Ce 3d spectrum of Co/CeO_2_ (Figure S3b), the peak at 882.7 eV was attributed to the Ce^4+^ state. The Ce^3+^ state shows a peak of 3d_5/2_ at ∼881 eV,^14^ while our catalyst did not. The presence of Ce^4+^ state was also confirmed by the strong peak at 917.0 eV, which is a characteristic satellite called u’’’.^15^ In the Co 2p spectra of these catalysts shown in Figure S3c, various valence states of Co ion were observed. The Co–O related peaks (Co^x+^) appeared at ∼781.2 and ∼786.5 eV, while the weak peak at ∼778.5 eV was ascribed to be a metallic Co state (Co^0^).^16^ From the peak area, the ratio Co^0^/Co^x+^ was calculated to be 0.43 for La/O_1.5_ or 0.18 for Co/CeO_2_. Table 1 also shows WFs estimated from the Kelvin probe. The WFs of Co/LaO_1.5_ and Co/CeO_2_ were 4.7 and 5.2 eV, respectively, which were smaller than that (5.3 eV) of metallic Co. This result indicates the enhanced Co–N interaction in Co/LaO_1.5_. The decrease in the WF of Co leads to the weakening of N–H bonds of NH_3_ absorbed on Co surfaces through electron donation^17^ and contributes to the cracking of NH_3_.

**Figure 3:**
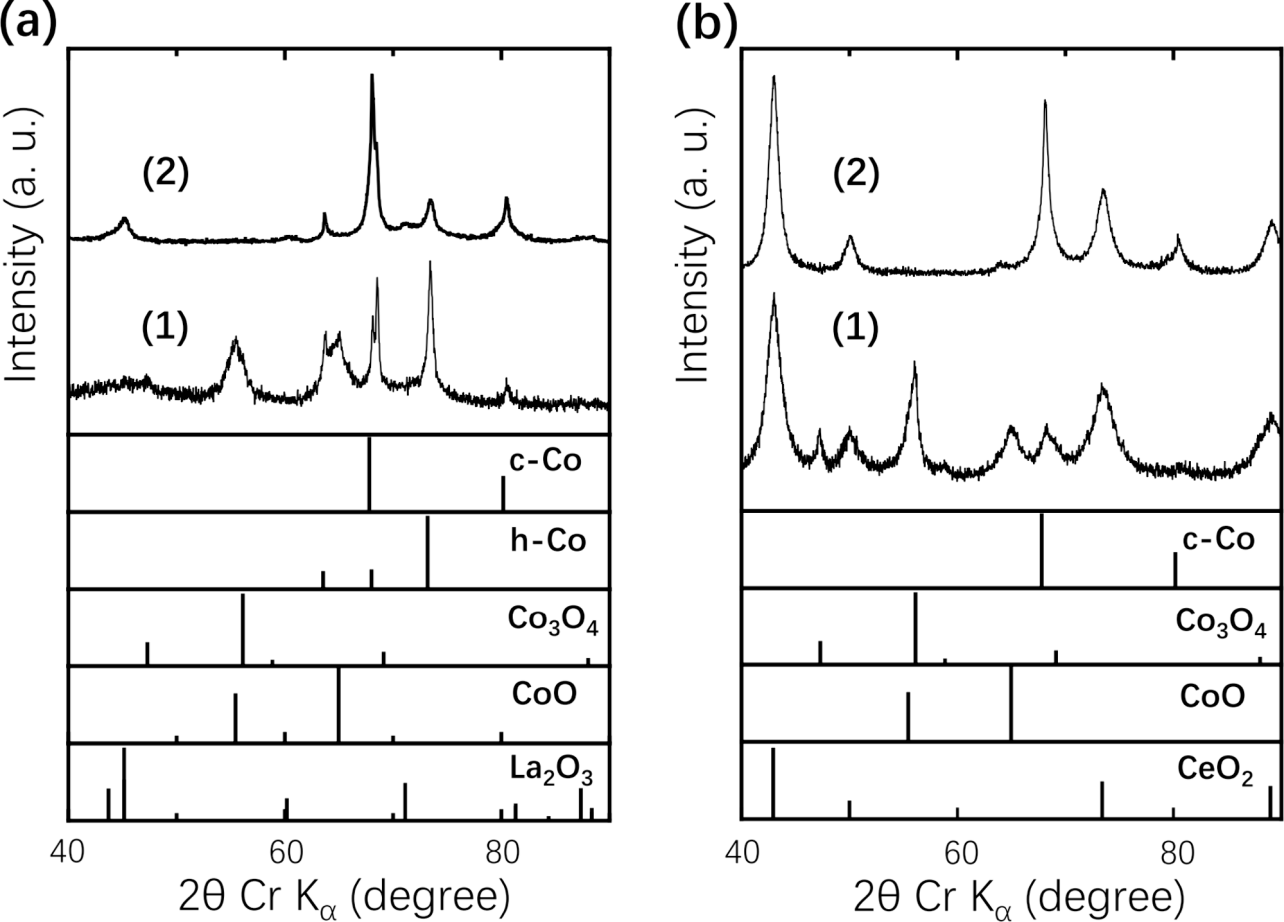
Powder XRD patterns of LaCo_13_ (a) and CeCo_3_ (b) catalysts. (1) Before NH_3_ test. (2) After NH_3_ test.

**Figure 4:**
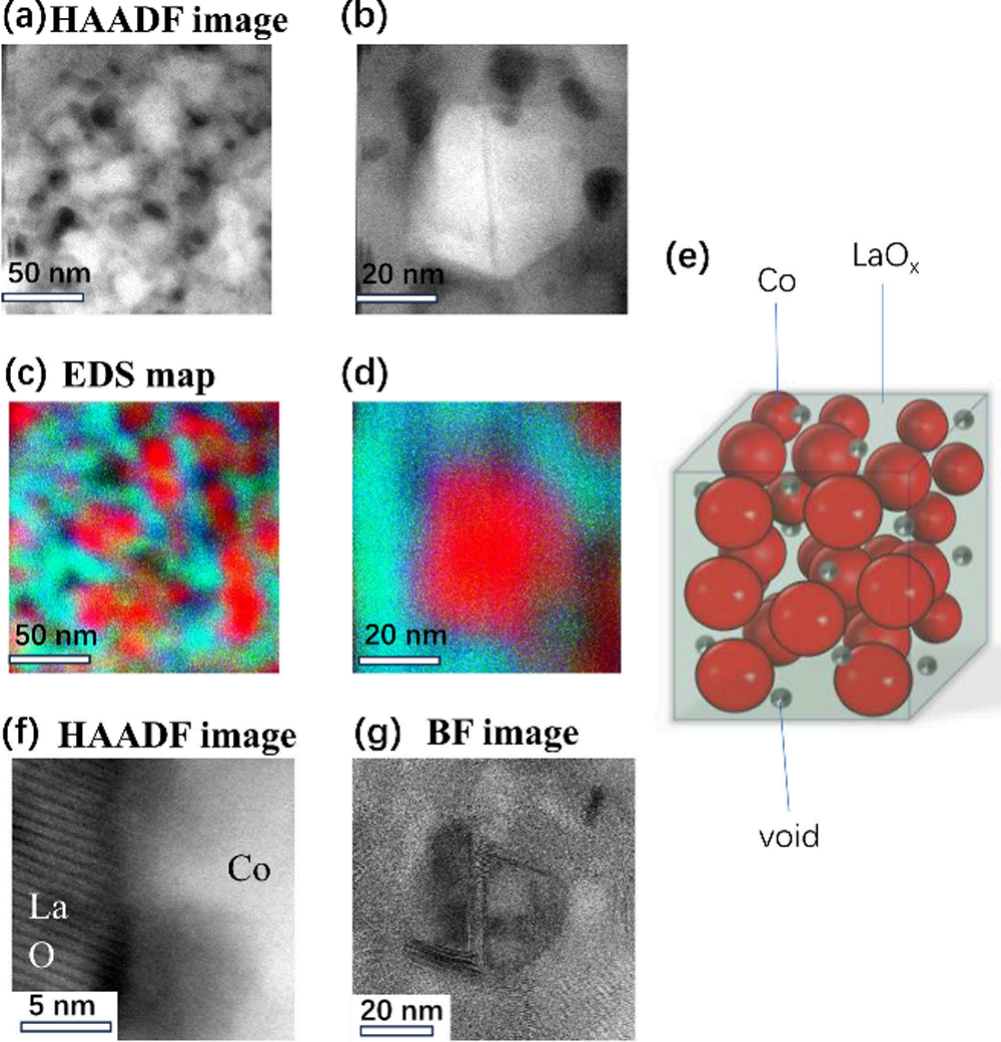
Nanostructures of Co/LaO_1.5_ catalyst after NH_3_ test. (a, b) HAADF images of Co/LaO_1.5_ nanocomposite. (c, d) The red, green, and blue areas in the EDX mapping images correspond to Co, La, and O, respectively. (e) Schematic of the nanostructure with Co particles enclosed by LaO_1.5_ matrix phase. (f) HAADF-STEM image of the Co/LaO_1.5_ interface. (g) BF image corresponding to (b).

**Table 2:** Crystallite sizes estimated by fundamental parameter method^b^

Catalyst^a^	Phase Volume weighted average diameter (nm)		
Co/LaO_1.5_	La_2_O_3_	c-Co	h-Co
	7(3)	17.2(2)	13.7(3)
Co/CeO_2_	CeO_2_	c-Co	
	8.05(5)	12.2(1)	

a After NH_3_ test

b No predominant strain was found.

The Co-rich compositions of Co/RO_*x*_ catalysts remind us inverse catalysts have a metal-rich composition.^18a,18b^ It has been often reported that increasing the area of the interface between the catalyst and the support enhances catalytic reaction in inverse catalysts. Here, we discuss the origin of the low WFs observed in our catalyst, which is expected to be the main factor affecting the observed catalytic activities. The low WF of Co/LaO_1.5_ indicates electron donation from the LaO_1.5_ matrix to the Co particles. Stoichiometric LaO_1.5_ does not commonly work as a semiconductor, because it is an insulator with a bandgap of ∼5 eV. However, the LaO_1.5_ phase has a smaller CS (∼10 nm), which is derived from the Co-rich composition. LaO_1.5_ with a smaller CS tends to be deficient in O at the surface of the particle. The presence of LaO_1.5-x_ at the surface is also consistent with the La 3d XPS spectrum (Figure S3a). In fact, it is reported on the basis of TEM observations and DFT calculations that CeO_2_ nanoparticles have Ce^3+^ ions at their surfaces, when the particle size is smaller than ∼10 nm.^19^ Recently, Hayami et al. showed by DFT calculations that metallic LaO_1.5-x_ with O-deficiency has an extremely small WF (∼2 eV), which originates from conduction electrons in the La 5d band.^20^ As shown in Figure 4f, the LaO_1.5_ phase adhered closely to the Co single crystal; consequently, a defective LaO_1.5_ region was formed in the interface. The observed low WF is realized by electron donation from the LaO_1.5-x_ matrix with an O deficiency to a Co particle in the interface. The enhancement of catalytic activity through the anion-deficient support in the interface has also been reported recently.^17,21^ We attempted to estimate the fraction of active Co species in the LaO_1.5_/(c-Co)_13_ composition using a rigid band model. When we used the magnetic density of states (DOS) of c-Co reported by Lizarraga et al.,^22^ the upshift of the Fermi energy of 0.6 eV (=5.3–4.7) is attained approximately through donation of 0.48 electrons to down-spin DOS. If we postulate a negatively charged Co^0.48–^ state, LaO_1.5_ must donate 6.2 electrons (=0.48 × 13) to Co_13_. LaO_*x*_ cannot have such a number of conduction electrons, indicating that only a portion of the Co ions among Co_13_ accept electrons from LaO_*x*_ and work as active species. This agrees well with the small metal surface area of Co/LaO_1.5_ estimated by the CO pulse chemisorption.

In summary, we investigated Co-based catalysts fabricated by the low-temperature thermal decomposition of R–Co IMs (R = Y, La, or Ce). (1) The Co/CeO_2_ nanocomposite prepared by the thermal decomposition of CeCo_3_ exhibited a superior catalytic activity of ∼30% at 400 °C for NH_3_ cracking. (2) In the Co/LaO_1.5_ catalyst derived from LaCo_13_, the Co nanoparticles of 10–30 nm in size are enclosed by the LaO_*x*_ phase. According to the Co-rich composition (Co/La = 13), the LaO_1.5_ phase inevitably has a smaller CS (∼10 nm), giving rise to the small crystallite size of LaO_1.5_. A low WF is realized for these catalysts by electron donation from the O-deficient LaO_1.5-x_ phase to the Co particles in the interface. (3) The separation into the metallic Co and LaO_1.5_ phases occurs. The LaO_1.5_ phase serves as a barrier for diffusion, preventing the grain growth of Co nanoparticles at high temperatures. (4) The thermal decomposition of IMs is a new route to designing unique nanostructures toward the development of catalytic systems.

## Supporting Information Available

Powder XRD pattern, TDS spectra, and XPS spectra (PDF)
