# Supplementary material for: Ammonia Decomposition Catalyzed by Co Nanoparticles Encapsulated in Rare Earth Oxide
Source: J Phys Chem Lett. 2025 Jan 14;16(3):796–801. doi: 10.1021/acs.jpclett.4c03309 (PMC11770756; doi:10.1021/acs.jpclett.4c03309)
Supplement: Supplementary file 1 — jz4c03309_si_001.pdf [file jz4c03309_si_001.pdf]

Supporting Information

Ammonia decomposition catalyzed by Co nanoparticles encapsulated in rare earth oxide

Hiroshi Mizoguchi,[a,§,*] Shunqin Luo,[a,§] Masato Sasase,[b] Masaaki Kitano,[b] and Hideo Hosono[a,b,*]

[a]Research Center for Materials Nanoarchitectonics (MANA),
National Institute for Materials Science (NIMS)
1-1 Namiki, Tsukuba, Ibaraki 305-0044, Japan

[b]MDX Research Center for Element Strategy, International Research Frontiers Initiative,
Institute of Science Tokyo, 4259 Nagatsuta, Midori-ku, Yokohama 226-8503, Japan

[§]These authors contributed equally to this work,
*Corresponding author footnote: mizoguchi.hiroshi@nims.go.jp.
hosono@mces.titech.ac.jp

(i)Synthesis

The starting materials were Co (Koujundo Chemical Lab., 99.9%), Y (Rare Metallic, 99.9%), La (Rare Metallic, 99.9%), Ce (Rare Metallic, 99.9%), $CoCl_2 \cdot 6H_2O$ (Wako, 99%), Y_2O_3 (Rare Metallic, 99.9%), La_2O_3 (Rare Metallic, 99.9%), and CeO_2 (ITEC, 99.9 %, 10±3 nm particle size). *R*–Co IMs (*R* = Y, La, or Ce) were prepared from a stoichiometric mixture of *R* and Co by arc melting on a water-cooled Cu hearth in Ar atmosphere. To fabricate Co nanoparticles, the thermal decomposition of IMs was attempted by low-temperature heat treatment. The heating conditions (350 °C, 12 h) under air were optimized by investigating the activity of the obtained catalyst. The process of preparing the catalysts is summarized in **Scheme 1**. As references, Co/RO_x catalysts were prepared by the impregnation method. $CoCl_2 \cdot 6H_2O$ was dissolved in distilled water and mixed with an RO_x supporter. The mixed solution was evaporated using a hot stirrer at 120 °C and subsequently calcined at 600 °C for 2 h in air.

(ii)Characterization

NH_3 cracking was performed in a fixed-bed plug-flow silica glass reactor (6 mm I.D.) placed in a furnace. Catalyst powder of 50 mg was placed on the silica wool in the reactor. Pure NH_3 was allowed to flow at a rate of 5–30 mL min^{-1} through a mass flow controller. A mass flow meter behind the reactor monitors the change in flow rate caused by NH_3 cracking, which was mathematically converted into a conversion ratio. Effluent gases were also analyzed using an

online gas chromatograph (GC-8A, Shimadzu, Japan) equipped with thermon–3000+KOH Sunpak columns and a thermal conductivity detector. The synthesized materials were identified by powder XRD (Miniflex600-Cr, Rigaku, Japan) using Cr K_α radiation. The structure refinements were performed by the Rietveld method as implemented in the TOPAS software package.[1] Profile fitting was performed by the convolution of emission profile, instrument component, and sample by a fundamental parameter method.[1] The sample part contained the contribution of both crystallite size and strain, which were proportional to $(\cos 2\theta)^{-1}$ and $\tan\theta$, respectively. The hydrogen or nitrogen content of the catalysts was estimated by thermal desorption spectroscopy (TDS). The Brunauer–Emmett–Teller (BET) surface areas were obtained from the BET plot at $P/P_0 < 0.3$. Co surface area was determined by CO pulse chemisorption (BELCAT-A, BEL, Japan) at 50 °C using a He flow of 50 mL min^{-1} and pulses of 0.03 mL (9.51% CO in He). Prior to CO pulse chemisorption, the catalysts were treated with flowing H_2 (50 mL min^{-1}) at 500 °C for 30 min and then with flowing He (50 mL min^{-1}) at 500 °C for 30 min to remove adsorbed H atoms from the reduced catalysts. A stoichiometry of Co/CO = 1 was assumed to calculate the metal dispersion. X-ray phototelectron spectroscopy measurements (XPS) were performed with a Quantes (ULVAC-PHY, Inc., Japan) with a monochromatic Al K_α X-ray source (hν = 1486.6 eV) operated at 15 kV. The workfunction (WF) was measured by Kelvin probe force microscopy (FAC–2, Riken, Japan) under ambient

pressure in a N_2-filled glovebox. Scanning transmission electron microscopy (STEM) and high-angle annular dark-field scanning transmission electron microscopy (HAADF–STEM) images were captured using a JEOL JEM–ARM 200F equipped with an EDX detector operating at 200 kV. To obtain cross-sectional STEM images, the prepared samples were cut to a thickness of 90 nm using a focused ion beam (JIB–4601F, JEOL, Japan) with a liquid gallium ion source. Here, a Peltier cooled sample stage (~223 K) was used to prevent damage to the samples.

Table S1. Catalytic performance of Co-based catalysts for NH_3 cracking under 1 atm.

Co wt%	supporter	promoter	WHSV ($mL\ g^{-1}\ h^{-1}$)	T (°C)	conv (%)	NH_3 decomposition rate ($mol_{NH3}\ g_{cat}^{-1}\ h^{-1}$)	ref
82	La_2O_3	-	12000	400	25	0.12	This work
51	CeO_2	-	12000	400	30	0.15	This work
58	CeO_2	Ba	9000	400	31	0.11	[2]
91	Al_2O_3	-	9000	400	27	0.10	[3]
8	La_2O_3	-	18000	400	6	0.05	[4]
20	MgO	La	22000	400	47	0.42	[5]
-	NC	-	30000	400	15	0.18	[6]
-	NC/SBA-15	La	30000	400	28	0.34	[7]
10	Al_2O_3	La	9000	400	11	0.04	[8]
17	Al_2O_3	-	24000	500	40	0.39	[9]
35	CNT	-	6000	500	75	0.18	[10]
~10	$Ce_{0.6}Zr_{0.3}Y_{0.1}O_{\sim2}$	-	6000	550	100	0.24	[11]

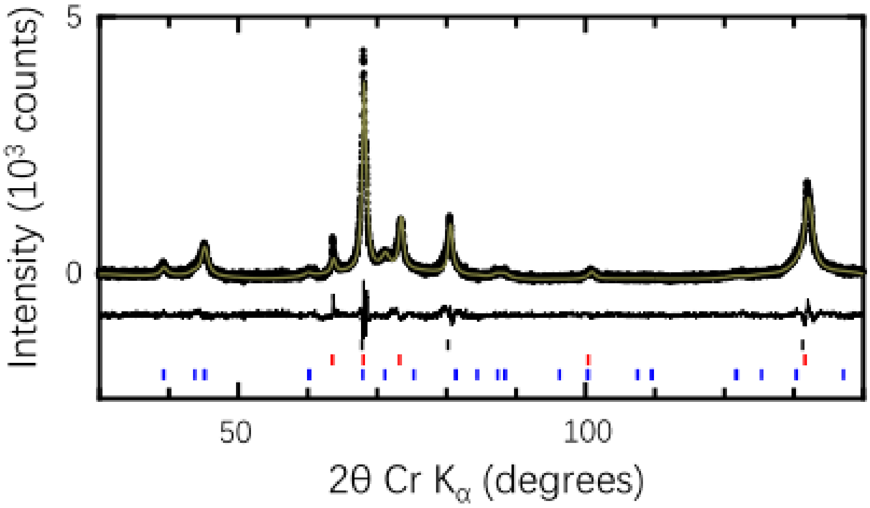

Fig. S1 XRD pattern of Co/$LaO_{1.5}$ after NH_3 test as measured (black dots) and refined by the Rietveld method (gray line). The vertical bars at the bottom show the calculated positions of the Bragg diffractions of c-Co (upper), h-Co (middle), and $LaO_{1.5}$ (lower). The obtained chemical composition was c-Co/h-Co/$LaO_{1.5}$ = 65.6/27.9/6.5 at%.

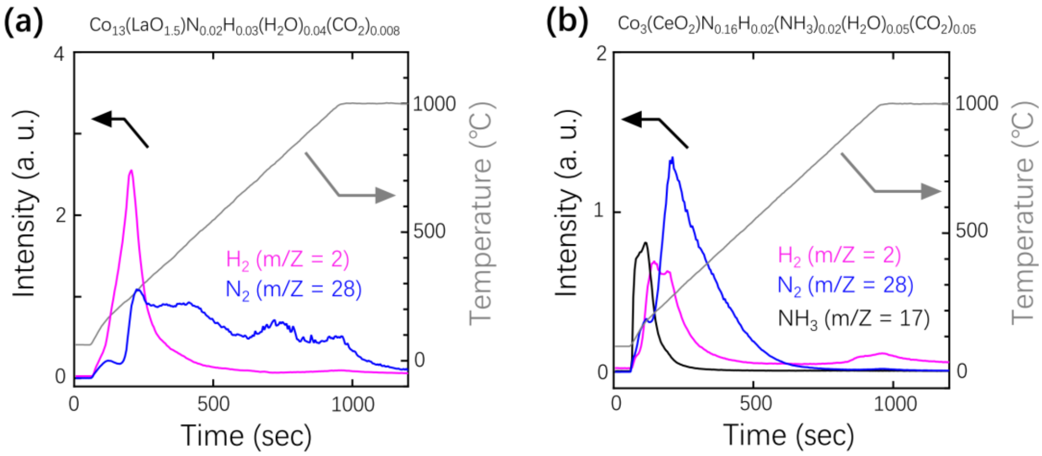

Fig. S2 TDS spectra at specific mass-to-charge ratios (m/z) of 2 (H_2), 17 (NH_3), and 28 (N_2) for **(a)** Co/$LaO_{1.5}$ and **(b)** Co/CeO_2 catalysts after NH_3 test.

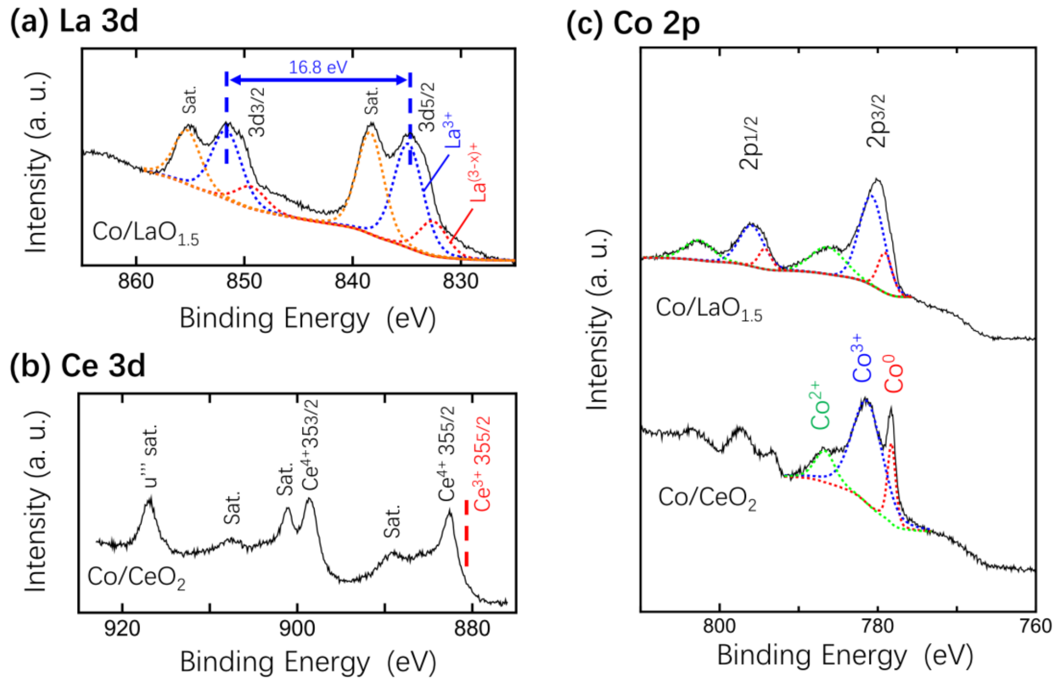

Fig. S3 XPS spectra of $Co/LaO_{1.5}$ and Co/CeO_2 catalysts. **(a)** La 3d, **(b)** Ce 3d, and **(c)** Co 2p.

References

1. Cheary, R. W.; Coelho, A. A., A Fundamental Parameters Approach to X-ray Line Profile Fitting. *J. Appl. Cyst.* **1992,** *25*, 109-121.

2. Morlanés, N.; Sayas, S.; Shterk, G.; Katikaneni, S.; Harale, A.; Solami, B.; Gascon, J., Development of a Ba–CoCe catalyst for the efficient and stable decomposition of ammonia. *Catal. Sci. Tech.* **2021,** *11* (9), 3014-3024.

3. Gu, Y.; Jin, Z.; Zhang, H.; Xu, R.; Zheng, M.; Guo, Y.; Song, Q.; Jia, C., Transition metal nanoparticles dispersed in an alumina matrix as active and stable catalysts for COx-free hydrogen production from ammonia. *J. Mater. Chem. A* **2015,** *3* (33), 17172-17180.

4. Xun, Y.; He, X.; Yan, H.; Gao, Z.; Jin, Z.; Jia, C., Fe- and Co-doped lanthanum oxides catalysts for ammonia decomposition: Structure and catalytic performances. *J. Rare Earths* **2017,** *35* (1), 15-23.

5. Hu, X.; Wang, W.; Jin, Z.; Wang, X.; Si, R.; Jia, C., Transition metal nanoparticles supported La-promoted MgO as catalysts for hydrogen production via catalytic decomposition of ammonia. *J. Energy Chem.* **2019,** *38*, 41-49.

6. Li, G.; Zhang, H.; Yu, X.; Lei, Z.; Yin, F.; He, X., Highly efficient Co/NC catalyst derived from ZIF-67 for hydrogen generation through ammonia decomposition. *Int. J. Hydrogen Energy* **2022,** *47*, 12882-12892.

7. Han, X.; Hu, M.; Yu, J.; Xu, X.; Jing, P.; Liu, B.; Gao, R.; Zhang, J., Dual confinement of $LaCoO_x$ modified Co nanoparticles for superior and stable ammonia decomposition. *Appl. Catal. B* **2023,** *328*, 122534.

8. Wang, W.; Fu, Y.; Wang, W.; Xiang, M.; Chen, G.; Su, Y.; Duan, J., Ammonia decomposition over La-doped Al_2O_3 supported Co catalyst. *Ceram. Inter.* **2024,** *50* (19), 36604-36614.

9. Gu, Y.; Xu, D.; Huang, Y.; Long, Z.; Chen, G., CO_x-free hydrogen production via ammonia decomposition over mesoporous Co/Al_2O_3 catalysts with highly dispersed Co species synthesized by a facile method. *Dalton Trans.* **2021,** *50* (4), 1443-1452.

10. Zhang, H.; Alhamed, Y. A.; Chu, W.; Ye, Z.; AlZahrani, A.; Petrov, L., Controlling Co-support interaction in Co/MWCNTs catalysts and catalytic performance for hydrogen production via NH_3 decomposition. *Appl. Catal. A* **2013,** *464-465*, 156-164.

11. Huang, C.; Li, H.; Yang, J.; Wang, C.; Hu, F.; Wang, X.; Lu, Z.; Feng, G.; Zhang, R., $Ce_{0.6}Zr_{0.3}Y_{0.1}O_2$ solid solutions-supported Ni Co bimetal nanocatalysts for NH_3 decomposition. *Appl. Surf. Sci.* **2019,** *478*, 708-716.
